# Effect of Density and Lethargy Duration in Prerelease Packaging of the Fruit Fly (Diptera: Tephritidae) Parasitoid, *Diachasmimorpha longicaudata* (Hymenoptera: Braconidae)

**DOI:** 10.1093/jisesa/ieaa004

**Published:** 2020-02-27

**Authors:** Jorge Cancino, Rubén Leal-Mubarqui, Roberto Angulo, Cesar Pérez, Lucy Tirado

**Affiliations:** 1 Programa Moscafrut, Metapa de Domínguez, Chiapas, México; 2 Servicios Mubarqui S. de R. L. de C. V., Cuidad Victoria, Tamaulipas, México; 3 Programa Moscamed, Tapachula, Chiapas, México

**Keywords:** parasitoid releasing, chilled adult, insect quality, fruit fly biological control

## Abstract

Different densities prerelease packing and times of lethargy in the fruit fly parasitoids *Diachasmimorpha longicaudata* (Ashmead) were evaluated in order to standardize the process of chilled insect technique for this species. Adults were kept at densities of 0.048, 0.072, 0.096, 0.120, and 0.144 parasitoids/cm^2^ before release in a México tower, where thermal lethargy was induced at a temperature of 2 ± 2°C for 45 min. Samples of parasitoids were collected to evaluate mortality, survival, fecundity, and flight capacity. All densities showed a similar mortality, both for males (ca. >10%) and females (ca. <7). There was no effect of density on survival and flight capacity in both sexes. On the other hand, fecundity increased with density, 1.66 sons/♀/day, similar to the control. We conclude that a density of 30,000 pupae per cage (0.144 parasitoids/cm^2^) is adequate for the massive prerelease packaging of the parasitoid *D. longicaudata*. Regarding the thermal lethargy period, 180 min under 2 ± 2°C conditions, considered as time for management, does not affect the survival, fecundity, and flight capacity of adults. The results obtained are of great utility to establish prerelease packaging parameters for *D. longicaudata* used in the biological control of Tephritidae fruit fly populations.

Augmentative parasitoid releases represent the most effective strategy to carry out fruit fly (Diptera: Tephritidae) biological control (Wong et al. 1991, Sivinski et al. 1996, Montoya et al. 2007). This technique requires an important technological development that involves the transport, management, and efficient distribution of the parasitoids. Different packaging techniques have been developed with the objective of favoring the transport of pupae over long distances with hypoxia (Cancino and López-Arriaga 2016), confinement conditions during emergence, and release (FAO/IAEA/USDA 2003, Cancino and Montoya 2006, Cancino et al. 2017). Currently, ground releases have been the common way to carry out fruit fly parasitoid artificial dispersion (Montoya et al. 2012). However, this method has its own limitations and an adequate distribution and dispersion of parasitoids in inaccessible areas has remained a challenge to the on-going control efforts.

Recently developed, the chilled adult technique represents an efficient procedure for packaging, handling, and releasing of different insect species (Hernández et al. 2009, Leal-Mubarqui et al. 2014). Particularly, the aerial release of sterile flies for the application of the Sterile Insect Technique (SIT) has relied on the release of lethargic insects, which are induced into such state by means of low temperatures. The advantages provided by the chilled adult technique may be important for application in fruit fly parasitoids. Furthermore, the release of chilled adults has been associated with an increase in flies’ dispersal (Hernández et al. 2009, Hernández et al. 2010, Andress et al. 2012, Leal-Mubarqui et al. 2014).

Relatively few studies have reviewed the effect of low temperatures on parasitoids. Exceptions include two references, which could be considered the first attempts to use thermal lethargy in fruit fly parasitoids for release. Sivinski, et al. (2000) and Baeza-Larios et al. (2002) showed that three species of fruit fly parasitoids maintain their important attributes after a process of dormancy. However, Montoya et al. (2012) and Cancino et al. (2017) reported mechanical damage in dormant adult parasitoids when prerelease packaging is massive. Apparently, the filamentous structures typical of Braconidae wasps with long antennae, legs, and ovipositors are sensitive to overcrowding conditions (Ruiz et al. 2009).

The most important advantage of the application of the ‘chilled adult’ technique is that it can be combined with aerial releases (Sivinski et al. 2000, Dowell et al. 2005), which could solve the limitations of distribution and dispersion of parasitoids with terrestrial releases. This becomes more important in areas with native vegetation located close to commercial orchards, which are generally difficult to access and act as refuge for many fruit fly pest species (Cancino et al. 2017).

The parasitoid *Diachasmimorpha longicaudata* (Ashmead) has been shown to represent the best option in the growing application of biological control of flies of the genus *Anastrepha* (Schiner) (Diptera: Tephritidae) (Sivinski et al. 1996, Montoya et al. 2000). Important technology for mass rearing and prerelease management has been developed for this species (Cancino and Montoya 2006, Cancino et al. 2017). The prerelease step in the mass insect management techniques is of utmost importance because it is when the final quality of the product (adult females) can be affected. Therefore, there has been a great interest in knowing the influence of factors such as density, food, and aromatherapy to stimulate copulation, among others (Barry et al. 2007, Perez-Staples et al. 2008, Haq et al. 2018). Important advances have been obtained with the application of SIT in wide-areas using chilled insect technique in fruit flies (Dowell et al. 2005, Hernández et al. 2010, Leal-Mubarqui et al. 2014, Orozco-Dávila et al. 2017). In the application of the ‘chilled adult’ technique, an initial standardization of the process is required so as to avoid problems derived from excessive cold as part of the lethargy induction.

In this study, the objective was to determine the effect of adult density on the prerelease mass packaging process and its consequences during lethargy induced by cold. In addition, we evaluated the effect of the duration of cold exposure on parasitoid quality parameters. Density and duration of exposure are basic parameters that must be established for the use of chilled insect in the packing for release of *D. longicaudata*. The results are of interest to optimize and standardized the management of this parasitoid in prerelease packaging using this chilled insect technique and its subsequent application in aerial release and to increase the advantages of a better distribution.

## Materials and Methods

### Biological Material

Pupae parasitized by *D. longicaudata* and third-instar *Anastrepha ludens* (Loew) (Diptera: Tephritidae) larvae (used as host) were obtained from the Moscafrut Program mass rearing, facility located in the Metapa de Domínguez Municipality, Chiapas, Mexico (SADER-IICA). The *D. longicaudata* colony (>350 generations) is maintained with a weekly production of 20–30 million pupae. The production of *A. ludens* exceeds 300 million pupae per week. Both mass rearings are performed based on a standard procedures established in the Moscafrut Plant (Cancino and Montoya 2006, Orozco-Dávila et al. 2017).

### Study Site

The packaging and induction of lethargy of *D. longicaudata* parasitoids were carried out in the Packaging Center of the Moscamed Program (SADER-SENASICA), located in the Cantón Leoncillos property at km. 19.8 of the Tapachula Highway-Puerto Madero, Chiapas, Mexico. The evaluation of quality parameters was performed at the Department of Biological Control of the Moscafrut Program.

### Prerelease Packaging

**Fig. 1.**
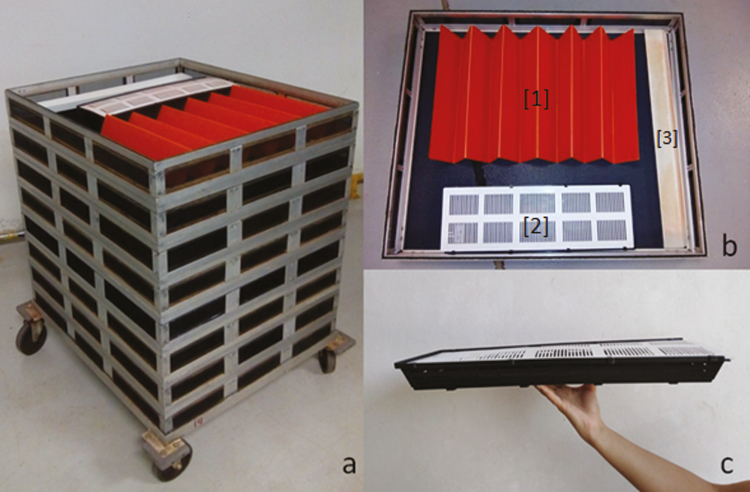
View of a level of Mexico tower: (a) Building a tower with the levels, (b) Parts of a level, [1] orange plastic-cardboard folded sheet, [2] Pupal Emergence Container (PEC) and [3] plastic container with food, and (c) A view of the PEC container for emergence of adults. Packaging Center, Moscamed Program, México.

**Fig. 2.**
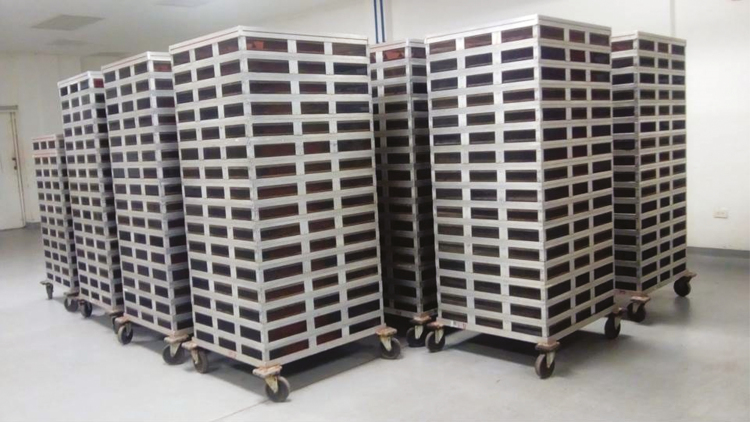
A lot of parasitoids packed in Mexico towers in the prerelease period. Packaging Center, Moscamed Program, México.

Pupae of *D. longicaudata* on development day 14 (1 d before emergence) were packed using the Mexico tower method (FAO/IAEA 2017). The Mexico tower is an aluminum structure (160 cm tall, 80 cm long, and 70 cm wide), with 16 levels which are stacked. Each level is a cage (10 cm tall, 80 cm long, and 70 cm wide) covered with a nursery-type mesh. This group of 16 cages is put on a metallic base (72 × 84 cm) with wheels for mobilization. Pupae were placed in a Pupal Emergence Container (PEC), which consists of a rectangular plastic container (50 × 15 × 5 cm) with lid. The lateral sides of PEC have longitudinal openings of 4 cm long and 3 mm wide. One PEC was placed inside of each tower level, together with an orange plastic-cardboard folded sheet (110 × 40 cm) to increase the resting surface for the emerged parasitoids, and a plastic container (60 × 6 × 2.5 cm) with 20 mg of a mixture of toilet paper soaked with honey (Figs. 1 and 2). Initially, the tower was maintained in a room at 26 ± 1°C and 60–70% relative humidity (RH) during 4 d. Adult emergence starts with males and 2 d later females; this process ends at 26°C in approximately 4 d (Montoya et al. 2012, Cancino et al. 2017). The emerged parasitoids went out through the longitudinal opening, leaving the empty puparium inside the PEC. The parasitoids fed on honey and used the folded sheets as resting areas. Once ended the emergence period (~70% adults emergence was completed), room temperature was reduced to 21 ± 1°C for 3 d, which avoids an increase in mortality while promoting copulation (Martínez et al. 1993). The prerelease packing is completed in 7 d.

### Induction to Lethargy

Adult parasitoids (6- to 8-d-old for males and 4- to 6-d-old females) kept in cages (levels of the tower) were transferred to cold chambers (12 × 6.20 × 3 m). As a first step, moisture was reduced with a dehumidifier (ICEDRY1400 Munters, México), using three 5-ton refrigeration units (SJH0501 M5C Bohn, México), the temperature was reduced to 15°C. Subsequently, the Mexico towers with parasitoids were introduced in the entered the cold room, and the temperature was reduced to approx. 2°C, which promotes parasitoid dormancy. It took 30 to 45 min and an average temperature of 2 ± 2°C to obtain the total lethargy state of the parasitoid lot.

### Collection of Biological Material

During the parasitoid lethargy, the levels of the México tower were separated progressively. The PEC, the plastic container whit food, and the folding sheet were removed from each level, taking out with a brush the dormant parasitoids on these attachments. Each level was turned over a collection table consisting of a metal funnel with a square mouth of 110 × 110 cm, and parasitoids were collected in a rectangular prism plastic container of 50 × 42 × 32 cm (PARC box). Afterward, we carried out the evaluation of effect of packed pupae density and lethargy duration, as explained in the following two subsections.

### Chilling Effect on the Packaging Density of Adult Parasitoids

Quantities of 10, 15, 20, 25, and 30 thousand pupae per level were packed. Considering a mean emergence rate of 68.57% (Quality Control Department, Moscafrut Program, Annual Report 2018), these numbers represented densities of 0.048, 0.072, 0.097, 0.121, and 0.145 adults/cm^2^, respectively. The treatments were distributed among the levels of the Mexico towers at random. Each one was included within a mass packaging event of 3 to 4 million pupae distributed in Mexico towers with a density of 15,000 pupae per level (approximately 0.072 adults/cm^2^, control density) (Ruiz et al. 2009). From the total adults collected from each treatment, a random sample of 30 ml of parasitoids (~5,800 parasitoids) freshly entered into lethargy was taken; subsequently, the samples were changed to a temperature of 26 ± 2°C, where the parasitoids regained their mobility and then evaluations of adult parasitoid quality were carried out. For each density, the tests of quality were: mortality during packaging, survival, fecundity, and flight capacity of adults. As a control treatment, parasitoids of the same lot were kept at 0.144 adults/cm^2^ under the same environmental and food conditions but in cages of 30 × 30 × 30 cm without being subjected to lethargy. Ten replicates per treatment were performed.

### Lethargy Duration

To determine the effect of the lethargy status duration, 90 cylindrical plastic containers (6.1 cm high and 5.8 cm in diameter) were placed with samples of 25 ml of dormant parasitoids in lethargy status (approximately 852 parasitoids) randomly chosen. Parasitoid samples were maintained at 2 ± 2°C for a variable lapse of time, ranging from 0 min (control) to a maximum of 180 min. Lethargy duration for each sample was randomly determined. After exposure to 2°C, insects were placed separately in plexiglass cages of 30 × 30 × 30 cm at 26 ± 2°C, where they recovered their normal mobility. These insects were later on used to perform the evaluations of quality described below (2.8). In this case, only survival, fecundity, and flight capacity were evaluated.

### Quality Evaluations

The measuring of each parameter was based on the quality procedures manual for fruit fly shipment (FAO-IAEA-USDA 2003) adapted to *D. longicaudata*, these were developed as follows:

#### Mortality

A 5 ml sample of dormant parasitoids (161.54 ± 3.10 parasitoids) was randomly taken, from which the total number of parasitoids classified by females and males were counted. When changing them to a temperature of 26°C, the number of live and dead parasitoids by sex could be differentiated, obtaining the percentage corresponding to each case based on the total.

#### Survival

A sample of 30 ♀ and 15 ♂ was placed inside a 30 × 30 × 30 cm plexiglass cage without water and food, and the number of dead parasitoids was recorded daily until the death of all individuals.

#### Fecundity

A sample of 30 ♀ and 15 ♂ was placed inside a 30 × 30 × 30 cm plexiglass cage. After 24 h, one hundred 8-d-old *A. ludens* larvae were exposed to parasitoids for 1 h in a Petri dish lid (9.1 cm in diameter and 0.9 mm height covered with a tricot cloth piece and secured with an elastic band). Exposure of host larvae was carried out for five consecutive days. From the 6th to 10th day when the parasitoid females have the highest fecundity (Lawrence et al. 1978, Meirelles et al. 2013). Subsequently, the larvae were separated from the larval diet by washing and kept in vermiculite in cylindrical plastic containers for 14 d at 26 ± 2°C and 60–80% RH. After offspring emergence, the number of offspring was related to the number of live females, and the average fecundity per day and total fecundity during the 5 d were obtained.

#### Flight Capacity

A sample of 100♀: 50♂ was placed in the bottom of a 3.5-inch black PVC tube (9 cm in diameter and 10 cm high), with neutral talc on the inside. The tubes were placed in 30 × 30 × 30 cm plexiglass cages where they were kept for 5 d. Each day, parasitoids that left the tube were removed (flyers), and the parasitoids that remained inside the tube (non-flyers) were counted. The number of flyers was related to the total number of parasitoids introduced; the results were reported as percentage of flying adults.

### Data Analysis

The number of dead and live parasitoids by sex after packaging were analyzed by applying a two-way analysis of variance (ANOVA). The factors were the densities and the number of parasitoids by sex. Box-Cox transformation was applied to solve problems of normality and homoscedasticity. The flight capacity was analyzed by ANOVA and by Tukey multiple comparison tests. Adult survival in the evaluation of densities as the duration of insect in lethargy was analyzed via a Log-rank test. Fecundity was considered for the offspring-per-day-average and was analyzed by a repeated-measures ANOVA. In this test, a two-way factorial design was applied, using as factors the days and the different densities. The average fecundity and flight capacity obtained at different periods of time for parasitoids in lethargy were analyzed with random samples taken at different time points in induced lethargy and were subjected to a regression analysis with respect to time. The statistical software JMP Version 5.7 was used.

## Results

### Chilling Effect on the Packaging Density of Adult Parasitoids

**Fig. 3.**
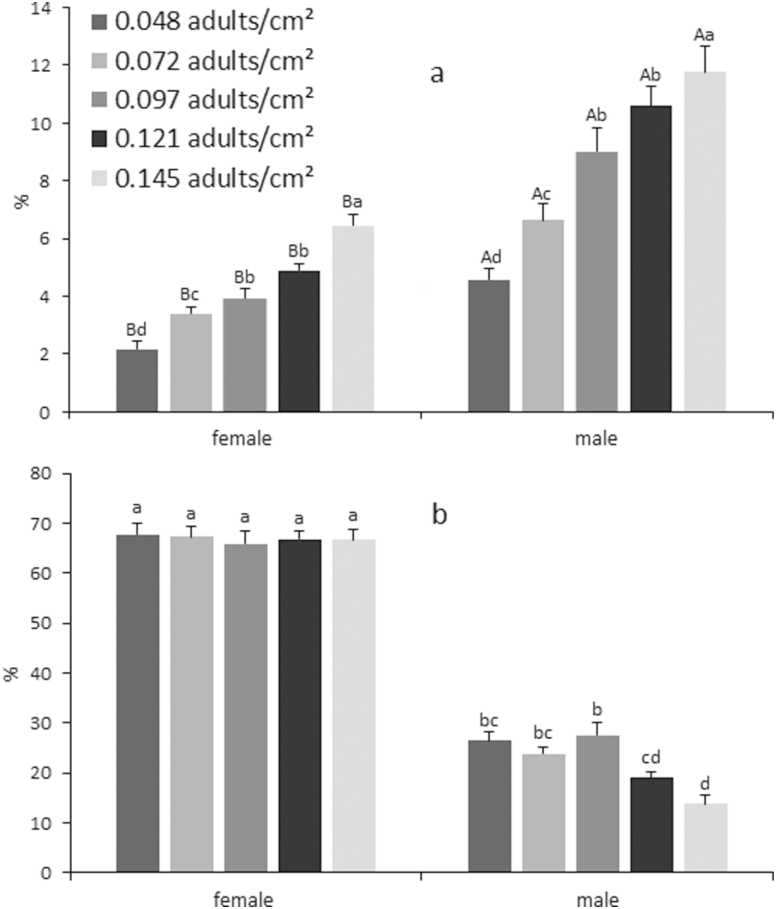
Percentages (average ± SE) of dead (a) and alive (b) females and males of *D. longicaudata* after prerelease packing in Mexico tower and submitted to lethargy with cold, using different densities of parasitoids (emerged from pupa) per level. Different letters above the bars in each group implicates statistic difference, (a) capital letters indicate difference between sexes, lowercase letters difference between densities, (b) lowercase letters indicate difference between sexes and densities. Two-way ANOVA and Tukey’s test.

The mortality of male and female parasitoids obtained after packing at different densities and subjecting them to lethargy increased significantly at higher density. The female mortality was always maintained at levels below 10%, including higher densities (*F* = 45.74; df = 4; *P* < 0.01). The increase of mortality was higher in males in comparison with females (*F* = 211.76; df = 1; *P* < 0.01). There was no interaction between densities and parasitoid sex (*F* = 2.13; df = 4; *P* = 0.08) (Fig. 3a). The number of live males had an inverse relationship with the density; therefore, the number of live adults was significantly lower at higher density. By contrast the number of live females remained in an average between 60 and 70% without presenting a significant difference among densities (density: *F* = 5.94; df = 4; *P* < 0.01; sex of parasitoid: *F* = 1132.67; df = 4; *P* < 0.01). The density and the sex of parasitoids were interactive factors in the number of live parasitoids (*F* = 10.11; df = 4; *P* < 0.01; Fig. 3b).

**Fig. 4.**
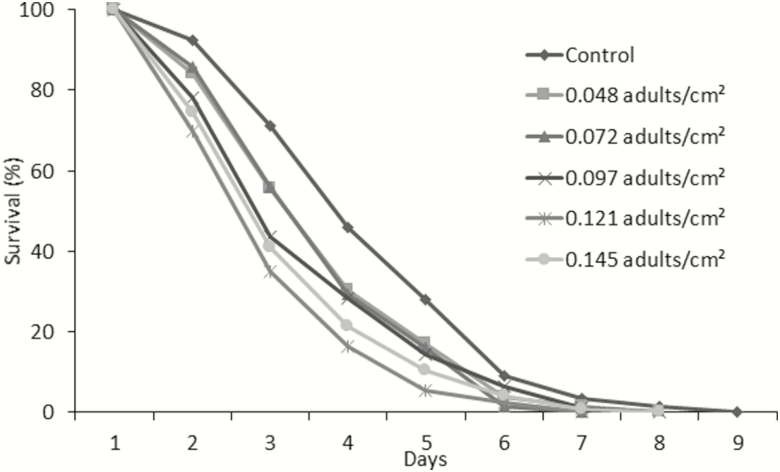
Longevity of adults of *D. longicaudata* without water and food maintained in Mexico towers in pre-releasing packing and submitted to lethargy by cold using different densities per level. There is no statistic difference among densities, Long-rank test.

Lethargy did not affect the survival of adult parasitoids at different densities. The results of the survival curves obtained showed no significant difference among them, including the control (*X*^2^ = 1.8; df= 5; *P* = 0.94) (Fig. 4). Similarly, the flight capacity of these parasitoids, which averaged approximately 60%, was not affected by lethargy duration. The averages obtained showed no significant difference among densities and the control (*F* = 2.09; df = 5,46; *P* = 0.09) (Table 1).

**Table 1.** Percentage (±SE) of flight capacity of *D. longicaudata* maintained in Mexico tower in prerelease packing and submitted to lethargy by cold using different parasitoid densities per level

Density of parasitoids (adults/cm^2^)	Flight capacity (%)
Control	61.52 ± 4.37
0.048	69.71 ± 3.78
0.072	66.13 ± 2.84
0.097	61.04 ± 4.07
0.121	61.23 ± 2.41
0.145	56.74 ± 1.44

There is no statistical difference among densities, ANOVA, and Tukey’s test.

**Fig. 5.**
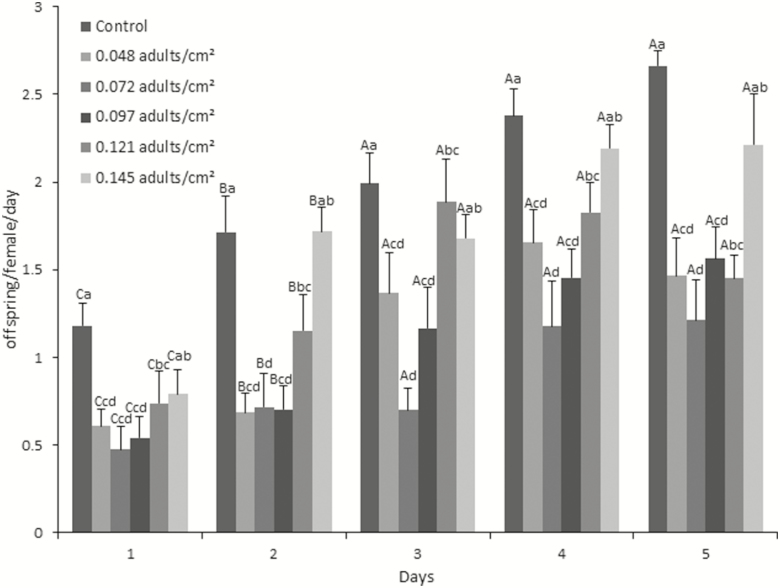
Daily fecundity (offspring/female/day ± SE) of females of *D. longicaudata* maintained in Mexico tower in prerelease packing and submitted to lethargy by cold, using different densities of parasitoids (emerged from pupa) per level. Different letters above the bars between days implicates statistic difference, capital letters indicate difference between days, lowercase letters difference between densities. Repeated measures ANOVA and Tukey’s test.

The fecundity of females was significantly different among days (*F* = 38.83; df= 4,179; *P* < 0.01) and among densities (*F* = 24.75; df = 5,179; *P* < 0.01), and there was no interaction between these factors (*F* = 1.68; df = 20,179; *P* = 0.16). The average fecundity values obtained from females confined at the highest density an average of 0.144 parasitoids/cm^2^ (30,000 pupae) and the control showed no significant difference during the 5 d of evaluation; at lower densities, the average fecundity values declined in adults subjected to lethargy with respect to the control. However, a significant decrease in the fecundity of parasitoids subjected to lethargy was obtained in the two first days, mainly when kept at low densities compared to the control without lethargy (Fig. 5).

### Lethargy Duration

**Fig. 6.**
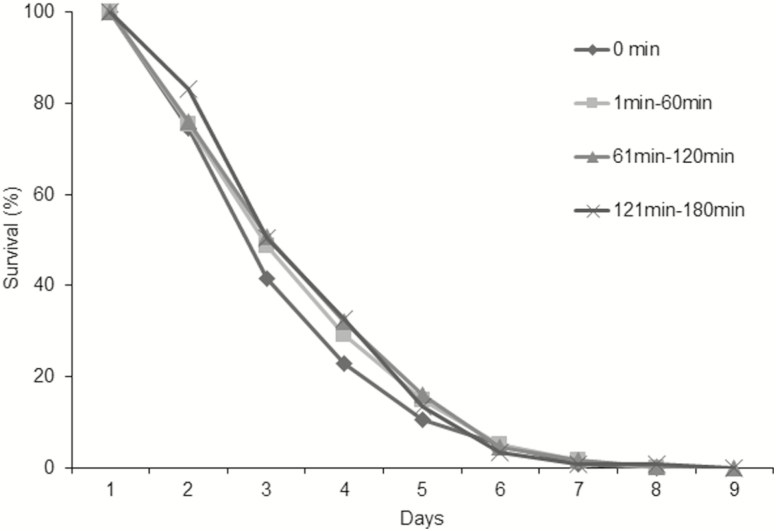
Longevity of adults of *D. longicaudata* without water and food and submitted to different periods in lethargy. There is no statistic difference among densities, Long-rank test.

**Fig. 7.**
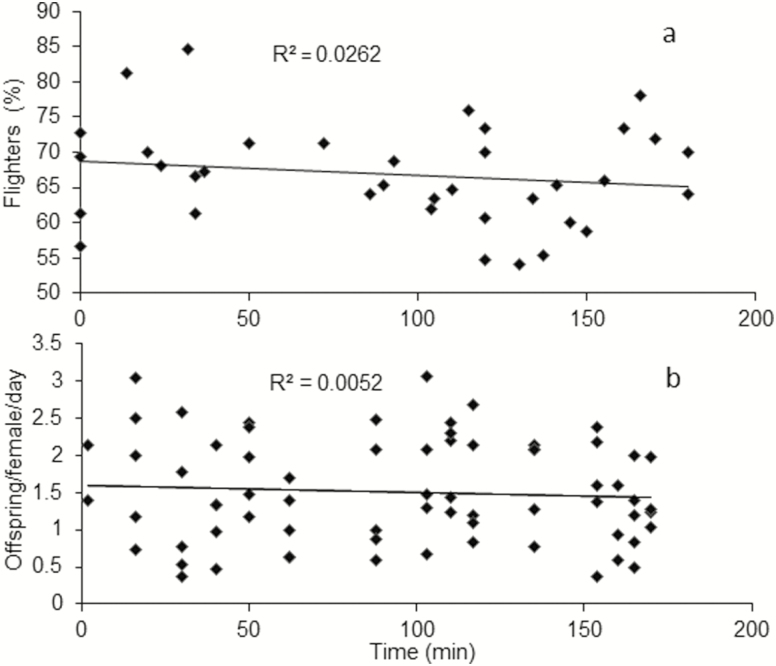
Relationship of flight capacity percentage (a) and fecundity (b) of parasitoids *D. longicaudata* submitted at different times of lethargy in a range from 0 to 180 min. There was no relationship between the flight capacity and fecundity with the time of chilling. Simple regression analysis for each parameter.

The lethargy period applied for parasitoids did not affect any of the evaluated parameters. Adult survival was not related to the dormancy duration after packing in a 0 to 180 min range (χ ^2^ = 0.35; df =3; *P* = 0.95) (Fig. 6). The averages of flying adults (*F* = 0.91; df=1,35; *P* = 0.3456, *R*^2^ = 0.03) and fecundity (*F* = 0.35; df = 1,68; *P* = 0.56, *R*^2^ = 0.005) had no relationship with the time of chilling (Fig. 7).

## Discussion

The increase in density per cage showed a direct effect on adult parasitoid mortality, although this negative effect was only less than 12% in males and 7% in females. These percentages of adult mortality can be considered as tolerable in the massive releases of this parasitoid. According to these results, it was possible to conclude that 0.145 adults/cm^2^ (~30,000 pupae) per level (individual cage) of the Mexico towers allow adequate management of parasitoids without presenting negative effects on the quality of adults. Neither, survival, fecundity, or flight capacity had a significant decrease. This density also favors fecundity, apparently as a result of stimulation and favoring a more successful copulation. Actually, little is known about the conditions that favor copulation in *D. longicaudata*. Sivinski and Webb (1989) report that there are characteristic sounds that stimulate copulation, which can be advantageous at high density. Copulation is an activity that is performed very quickly, right after females emerge, which is stimulated by a sexually mature male that has emerged 2 d in advance (Carrión 2000). The presence of repeated copulations and male competitive activity that involves much friction among individuals has also been reported (Carrión 2000). It is usually considered that greater copulation activity stimulates oviposition in *D. longicaudata* (Lawrence et al. 1978).

The density of adults at each Mexico tower level for prerelease packaging did not show a correspondence with the percent of alive females; the negative effect occurred in males only at higher density. The survival period of *D. longicaudata* males is short compared to that of females, they emerge 2 d before the females, copulate, and their average survival does not exceed 5 d, at which time the adults are kept in prerelease packaging (Viscarret et al. 2006, Meirelles et al. 2013). The increase in mortality of males could be a result of greater stress at higher densities and the possible reproduction activation (Paranhos et al. 2008, Wanjberg et al. 2012). The application of *D. longicaudata* for the biological control of *Anastrepha* spp. is by augmentative releases (Montoya et al. 2007), where the high male mortality is not a problem, as mated females are the ones that play the fundamental role, given that the search for a host and oviposition on it depend on them.

On the other hand, the survival of females and males, flight capacity, and average fecundity were not affected by cold treatment. All parameters were according with the quality parameters obtained in other evaluations (Gonzales et al. 2007, Lopez et al. 2009). However, in the way fecundity was presented, it could be a subject of future research. It was observed that the females subjected to dormancy at the end had an average fecundity similar to the control, but the fecundity per day was significantly lower in the first days. Usually, the sinovigenic parasitoids are sensitive to temperature changes. This implicates, in many occasions, severe alterations in their physiology of reproduction (Reznik et al. 2009, Redolfi and Campos 2010). Particularly, dormancy delays some physiological activities related to reproduction. After a period of dormancy, females may require a recovery period to obtain their normal reproductive capacity (MacMillan et al. 2012), which could have negative impacts on augmentative releases, where parasitoid actions are supposed to be rapid and suppressive in such a way that the pest population can be kept at low levels and not let them rise. A decrease in this respect can be risky, so immediate effects of cold on fecundity deserve further study.

With respect to lethargy duration, a period of 180 min was not detrimental to the quality parameters of survival, flight, or fecundity. Normally, this time is used in the handling of collection, prerelease, and release by plane. The data indicate that *D. longicaudata* can resist this process, which has been reported for other closely related fruit fly parasitoids in previous evaluations (Sivinski et al. 2000).

The results indicate that a density of 0.145 adults/cm^2^ can be managed in the Mexico tower levels and that up to 180 min in lethargy does not cause problems in the quality of female parasitoids. However, two important questions remain. Firstly, it is necessary to remember that these are activities of mass management of biological material, where the effects of high overcrowding are important to consider. Therefore, it is highly recommended to conduct post-mass release evaluations where there are more conclusive data on the effect of density and cooling. Secondly, it is necessary to reconsider the damage with a high priority parameter (Ruiz et al. 2009, Montoya et al. 2012). In our work, it was not a visible problem since an average of less than 5% of damage to antennae, and zero in other structures, was observed, which was attributed to the low humidity used in the process (average of 24.0% RH), which has been mentioned by different authors as a determining factor (Leopold 1998, MacMillan et al. 2015). According to Dowell et al. (2005), the reduction of moisture has been a constant suggestion in the mass releases of different insect species using the chilled adult technique.

Establishing parameters for the use of the chilled adult technique in fruit fly parasitoids is an initial phase to standardize a prerelease packaging process and to propose a more efficient field release method. It is mostly suggested that the biological control of this pest be performed through augmentative releases in wild areas. Occasionally, the topography of these areas prevents a uniform distribution of parasitoids. Aerial release using the cold adult technique might allow full access to such areas and, therefore, a better distribution of parasitoids, which is expected to significantly improve the control of pest populations in wild areas, preventing invasion of commercial orchards in a regional, area-wide, control program.
